# Effects of Dietary Glycine Supplementation on Reproductive Outcomes and Early Piglet Growth in Local Black Gilts: Associations with Placental Traits and Maternal Gut Microbiota–Metabolome Profiles

**DOI:** 10.3390/ani16192995

**Published:** 2026-09-23

**Authors:** Xingfu Chen, Yuening Xing, Dexin Yuan, Mengxian Chen, Ning Xu, Tiansheng Ma, Keming Huang, Suo Li

**Affiliations:** College of Animal Science and Technology, Jilin Agricultural University, Changchun 130118, China; reaixuexi2025@163.com (X.C.); ningww0913@163.com (Y.X.); dexin_xiey@163.com (D.Y.); 19855004356@163.com (M.C.); xiaoyangjueqi@163.com (N.X.); 18186310154@163.com (T.M.); huangkeming132@163.com (K.H.)

**Keywords:** local black pig, gilt, glycine, reproductive performance, placenta, gut microbiota, metabolomics

## Abstract

Dietary glycine may influence reproductive outcomes, placental development, and early piglet growth in gilts. Sixty local black gilts were randomly assigned to a basal diet (CON), an isonitrogenous control supplemented with 1.19% L-alanine (ALA), or 1.0% glycine (GLY; *n* = 20 per group) from 30 d before mating. After pregnancy confirmation, 10 pregnant gilts per treatment were randomly selected according to the prespecified design for follow-up to farrowing. Pregnancy rate differed among treatments (*p* = 0.004) and was higher in GLY than in CON. Compared with both controls, GLY had greater live-born litter weight at birth (*p* < 0.001), a lower proportion of low-birth-weight piglets (*p* = 0.010), and greater litter weight at 28 d (*p* < 0.001). GLY also had greater placental vascular densities and higher expression of *KDR*, *NOS3*, and several nutrient-transport-related genes. At gestation day 109, GLY was associated with treatment-related gut microbial differences and significant enrichment of fecal purine metabolism, with higher normalized abundances of putatively annotated adenosine, guanosine, and cGMP than in both controls. Overall, glycine supplementation was associated with improved selected reproductive and early piglet growth outcomes and altered placental and metabolic features.

## Simple Summary

The nutritional status of local black gilts can influence reproductive performance and early piglet development. This study evaluated 1.0% dietary glycine supplementation initiated 30 d before mating. After pregnancy confirmation, 10 pregnant gilts per treatment were randomly selected according to the prespecified study design to continue their assigned treatment until farrowing and for subsequent follow-up. Compared with a basal-diet control and an isonitrogenous L-alanine control, glycine supplementation was associated with greater live-born litter weight at birth, a lower proportion of low-birth-weight piglets, and greater litter weight at 28 d. Pregnancy rate was also higher than in the basal-diet control. Glycine supplementation was accompanied by greater placental vascular density and altered expression of genes related to vascular function and nutrient transport. In late gestation, treatment-associated differences were observed in the maternal gut microbiota and fecal metabolome, and purine metabolism—a pathway involved in nucleotide synthesis—remained significantly enriched after multiple-testing correction. Overall, 1.0% glycine supplementation was associated with improved selected reproductive and early piglet growth outcomes and altered placental traits in local black gilts.

## 1. Introduction

Chinese local black pigs are important indigenous genetic resources with value for high-quality pork production and germplasm conservation. Improving reproductive efficiency in gilts and reducing adverse piglet birth outcomes are important for the efficient use of these local resources. Previous comparisons between local black pigs and commercial breeds have identified placental molecular differences associated with reproductive performance [1]. During the first pregnancy, gilts must support their own growth while meeting the nutrient demands of embryo implantation, placental formation, and fetal development. Consequently, they can be sensitive to dietary amino acid supply and maternal metabolic status [2,3]. Amino acid and energy intake during late gestation can influence maternal body-weight change, piglet birth weight, and preweaning survival, with responses depending on nutrient supply and parity [4]. The placenta is the principal interface for maternal–fetal exchange, and its vascular development and nutrient-transport capacity are important determinants of fetal nutrient supply and growth [3,5,6]. Therefore, appropriate amino acid nutrition during gestation may help improve reproductive and piglet birth outcomes in local black gilts.

Glycine is the structurally simplest proteinogenic amino acid and is traditionally classified as nutritionally nonessential. Under physiological states with high nutrient demand, however, endogenous glycine synthesis may be insufficient to meet whole-body and tissue-specific metabolic requirements [7,8,9]. Stable-isotope studies in humans indicate that glycine may become conditionally indispensable during late pregnancy [10]. Whether a comparable limitation occurs in pregnant sows, and whether additional glycine can improve reproductive outcomes or placental vascular and nutrient-transport-related traits, remains unclear.

Glycine is an important substrate for protein and collagen synthesis [8,11] and participates in glutathione synthesis, one-carbon metabolism, de novo purine nucleotide synthesis, immune regulation, and nitrogen metabolism [9,12]. Because glycine contributes directly to de novo purine nucleotide synthesis, its availability may influence related nucleotides and metabolites [9]. Adenosine, a purine-related nucleoside, participates in vascular regulation and angiogenesis [13]. In pregnant sows, supplementation with 5′-adenosine monophosphate or adenosine can increase placental vascular density and improve neonatal piglet vitality [14]. Postnatal studies also show that dietary glycine supplementation can improve growth in piglets with intrauterine growth restriction [15], with accompanying changes in mTOR signaling, glutathione availability, and creatine availability [16,17,18]. These findings support biological roles for glycine in processes relevant to fetal growth and placental function, but direct evidence from glycine supplementation during gestation remains limited.

Dietary glycine supplementation may also be accompanied by changes in maternal gut microbiota and fecal metabolomic profiles. The maternal gut microbiota changes substantially during pregnancy and is associated with host metabolism, immune regulation, and pregnancy outcomes [19,20,21]. Nutritional interventions during sow gestation can modify fecal microbial composition and metabolic profiles. For example, N-carbamylglutamate supplementation altered fecal microbiota and serum metabolites in primiparous sows and was accompanied by changes in reproductive performance [22]. Whether glycine supplementation produces comparable maternal microbial and fecal metabolic signatures, and how such signatures relate to placental and reproductive phenotypes, remains unknown.

Therefore, this study used local black gilts and included an L-alanine isonitrogenous control to account for the effect of additional nitrogen supply. Glycine was supplemented at 1.0% from 30 d before mating to farrowing. We evaluated reproductive outcomes, piglet birth and early growth, and placental vascular and nutrient-transport-related traits. Maternal gut microbiota and fecal metabolomic profiles were also characterized to identify biological features associated with the phenotypic responses. We hypothesized that dietary glycine supplementation would improve selected reproductive and early piglet growth outcomes and would be accompanied by changes in placental vascular and nutrient-transport-related traits.

## 2. Materials and Methods

### 2.1. Animals, Experimental Design, and Husbandry

A completely randomized design was used. Experimental animals were selected from a herd of local black gilts raised at Jilin Shuangtian Ecological Agriculture Co., Ltd. (Liaoyuan, China). Sixty healthy gilts, approximately 9 months of age and with an initial body weight of approximately 110 kg, were selected with similar body condition and randomly allocated by drawing lots to three treatments (*n* = 20 per treatment): a basal-diet control (CON), an L-alanine isonitrogenous control (ALA), and a glycine-supplemented group (GLY). Each gilt was housed individually and received its assigned treatment individually; therefore, the gilt was the experimental unit. The initial sample size of 20 gilts per treatment was determined by the number of eligible animals available on the commercial farm, and no a priori power analysis was performed.

All 60 gilts were included in the pregnancy-rate analysis. Before treatment allocation, the study protocol specified that 10 pregnant gilts per treatment would be randomly selected after pregnancy confirmation for subsequent follow-up. Pregnancy was confirmed in 12 CON, 16 ALA, and 20 GLY gilts, after which 10 pregnant gilts from each treatment were randomly selected by drawing lots to continue the assigned dietary treatment until farrowing and for subsequent analyses. The remaining pregnant gilts were not included in the follow-up cohort; experimental amino acid supplementation was discontinued for the nonselected ALA and GLY gilts, and none of the nonselected gilts were systematically followed for downstream outcomes. At baseline, the gilts subsequently selected for follow-up had body weight, age, and body condition similar to those of the other pregnant gilts within the corresponding treatment group. No formal blinding was implemented during treatment administration, outcome assessment, or statistical analysis.

According to the farm breeding schedule, gilts were assigned to a planned breeding cohort. Amino acid supplementation began 30 d before the planned breeding period and, for gilts selected after pregnancy confirmation, continued until farrowing. Gilts were fed twice daily at 07:00 and 14:00. Daily feed allowances were approximately 1.75, 1.90, and 2.60 kg/gilt during D-30 to D59, D60 to D94, and D95 to farrowing, respectively. Farm staff visually monitored feeding behavior, apparent feed refusals, and general health daily.

To synchronize estrus before breeding, each gilt received altrenogest (Virbagest^®^, Virbac, Carros, France; 4 mg/mL) orally at 20 mg (5 mL) once daily for 18 consecutive days. After altrenogest withdrawal, estrus was monitored according to routine farm procedures, using the back-pressure response as the criterion for estrus detection. Gilts were then bred by artificial insemination. The same synchronization, estrus-detection, and artificial-insemination procedures were applied to all treatment groups, and semen was obtained from local black boars maintained on the same farm. The day of mating was designated as gestation day 0 (D0).

On gestation day 107, gilts were moved to individual farrowing crates. Glycine and L-alanine supplementation was discontinued at farrowing, and all treatment groups received the same lactation diet. Feed allowance was gradually increased after farrowing, and sows had ad libitum access to feed and water during lactation. Piglets were not cross-fostered, redistributed among litters, or standardized to a uniform litter size after birth. The experimental design and major sampling timeline are shown in Figure 1.

### 2.2. Experimental Diets and Amino Acid Supplementation

The basal diets were commercial complete feeds routinely used at the experimental pig farm, with gestation and lactation diets provided according to the corresponding production stage. All treatment groups received the same basal diet and feeding regimen within each production stage. Ingredient composition was obtained from the original diet formulation records of the experimental pig farm, and the calculated nutrient values are presented in Table 1. Manufacturer-provided premix specifications were used to calculate the vitamin–mineral contributions of the premixes to the complete diets.

Glycine and L-alanine were purchased from Hebei Huaheng Biotechnology Co., Ltd. (Hengshui, Hebei, China), with purities of 98.9% and 99.32%, respectively. The GLY group received supplemental glycine equivalent to 1.0% of the total daily feed allowance, the ALA group received 1.19% L-alanine as an isonitrogenous control, and the CON group received only the basal diet. The glycine supplementation level and the isonitrogenous L-alanine level were selected with reference to a previous pig study [23]. The L-alanine proportion was calculated from the nitrogen contents of the two amino acids to provide an equivalent amount of supplemental nitrogen.

Accordingly, the planned supplemental amounts were 17.5, 19.0, and 26.0 g glycine/gilt/d and 20.8, 22.6, and 30.9 g L-alanine/gilt/d during D-30 to D59, D60 to D94, and D95 to farrowing, respectively. The entire daily supplement was mixed with the 07:00 morning meal in the individual feed trough and provided once daily.

### 2.3. Sample Collection and Data Recording

Pregnancy was diagnosed at approximately 28 d after mating by transabdominal ultrasonography using a portable veterinary B-mode ultrasound unit equipped with a 3.5 MHz probe. Pregnancy rate was calculated based on the 20 gilts initially allocated to each treatment.

Fresh fecal samples were collected on gestation days 30 (D30) and 109 (D109). Immediately after spontaneous defecation, feces were collected from the interior of the fecal mass without contact with the floor, urine, or other contaminants; placed on dry ice; and transferred to −80 °C for storage. At D30, 10 fecal samples were collected per treatment. The same pre-sequencing DNA quality-control criteria were applied to all samples; one sample from each group was excluded because its DNA concentration or purity did not meet the requirements for subsequent 16S rRNA gene sequencing, leaving nine samples per group for analysis. At D109, fecal samples from all 10 gilts per treatment were used for 16S rRNA gene sequencing and untargeted metabolomic analysis.

At farrowing, total born, born alive, and stillborn piglets were recorded for each litter. All live-born piglets were weighed individually within 1 h after birth, and live-born litter weight at birth and mean individual birth weight were calculated. Piglets with a birth weight <0.9 kg were classified as low-birth-weight piglets using a prespecified operational threshold; comparable cutoffs of <0.9 kg and <1.0 kg have been used in previous Chinese sow and indigenous pig studies, respectively [24,25]. Piglets were weaned at 28 d of age, and litter weight at 28 d was recorded for each litter.

After farrowing, five of the 10 sows included in subsequent analyses in each treatment were randomly selected for placental sampling. One intact placenta was randomly selected from each sow. Five tissue pieces, each approximately 1 × 1 cm, were excised from each selected placenta at approximately 10 cm from the umbilical cord attachment site while avoiding areas of necrosis, hemorrhage, calcification, and grossly visible large blood vessels. The collected tissue was used for histological examination and gene expression analysis.

### 2.4. Placental Histology and Quantification of Vascular Density

Placental tissue samples for histological analysis were immediately fixed in 4% paraformaldehyde for 24 h. Samples then underwent routine dehydration, clearing, paraffin embedding, sectioning at 4 μm, and hematoxylin and eosin (H&E) staining. Histological observation and image acquisition were performed using an Olympus light microscope.

Placental vascular density was quantified using ImageJ software (version 1.53t; National Institutes of Health, Bethesda, MD, USA) [26]. For each sow, five non-overlapping fields of placental stroma and five non-overlapping fields of placental folds were randomly selected and analyzed at 40× and 400× magnification, respectively. Fields containing section-folding artifacts, tissue damage, necrosis, or large vessels > 100 μm in diameter were avoided. Placental stromal vascular density (PSVD) was expressed as vascular area relative to total stromal tissue area (%). Placental fold vascular density (PFVD) was expressed as the number of capillaries per unit tissue area in the placental folds. Measurements from all fields for each sow were averaged and treated as one biological replicate for statistical analysis.

### 2.5. RNA Extraction and Quantitative Real-Time PCR

Placental samples for gene expression analysis were rapidly frozen on dry ice and stored at −80 °C. For analysis, placental tissue was ground in liquid nitrogen, and total RNA was extracted using the TransZol Up Plus RNA Kit (TransGen Biotech, Beijing, China). RNA concentration and purity were measured using a NanoDrop One spectrophotometer (Thermo Fisher Scientific, Waltham, MA, USA), and RNA integrity was assessed by 1.2% agarose gel electrophoresis. A total of 1 μg RNA was reverse-transcribed using TransScript Uni All-in-One First-Strand cDNA Synthesis SuperMix for qPCR (TransGen Biotech, Beijing, China).

Quantitative real-time PCR was performed using PerfectStart Green qPCR SuperMix (TransGen Biotech, Beijing, China) on a CFX Real-Time PCR Detection System (Bio-Rad, Hercules, CA, USA). The angiogenesis- and vascular-function-related genes *VEGFA*, *NOS3*, and *KDR* and the nutrient-transport-related genes *GLUT3*, *GLUT4*, *SNAT1*, *SNAT2*, *FATP4*, and *CD36* were analyzed, with *ACTB* (β-actin) used as the reference gene. Three technical replicates were performed for each biological sample. The CON group was used as the calibrator, and relative mRNA expression was calculated using the 2^−ΔΔCt^ method. Primer sequences are provided in Table S1.

### 2.6. 16S rRNA Gene Sequencing and Bioinformatics Analysis

Fecal samples collected on gestation days 30 (D30) and 109 (D109) were subjected to 16S rRNA gene sequencing by Shanghai Personal Biotechnology Co., Ltd. (Shanghai, China), with *n* = 9 per treatment at D30 and *n* = 10 per treatment at D109. Microbial DNA was extracted from feces using the MagBeads FastDNA Kit for Soil (MP Biomedicals, Irvine, CA, USA), and DNA concentration and quality were assessed using a NanoDrop NC2000 spectrophotometer and agarose gel electrophoresis.

The V3–V4 region of the bacterial 16S rRNA gene was amplified using primers 338F (5′-ACTCCTACGGGAGGCAGCA-3′) and 806R (5′-GGACTACHVGGGTWTCTAAT-3′). PCR products were purified, quantified, pooled in equimolar amounts, and used for library construction, followed by 250 bp paired-end sequencing on an Illumina NovaSeq 6000 platform (Illumina, San Diego, CA, USA).

Raw sequences were processed using QIIME 2 [27]. Quality filtering, denoising, paired-end merging, and chimera removal were performed using DADA2 [28], yielding amplicon sequence variants (ASVs) and an abundance table. Taxonomic annotation was performed using the Greengenes2 reference database (release 2022.10; gg2_2022_10) [29]. Alpha diversity was evaluated using the Chao1, observed species, Shannon, and Simpson indices. Beta diversity was assessed by principal coordinate analysis (PCoA) based on Bray–Curtis distances.

LEfSe analyses were performed separately at D30 and D109 to identify treatment-associated taxa at each sampling time [30], using an LDA score > 2.0 and nominal *p* < 0.05 as the screening criteria. Because amino acid supplementation began at D-30, D30 was considered a post-treatment early-gestation sampling point rather than a pretreatment baseline. The D30 and D109 analyses were therefore interpreted as separate cross-sectional comparisons, and no within-animal temporal treatment effect was inferred. For downstream candidate-genus screening and microbiota–metabolite integration, analyses were focused on D109 because this late-gestation sampling point was closest to farrowing and the subsequent placental and reproductive assessments. At D109, genus-level relative abundances were compared pairwise between groups using two-tailed independent-samples *t*-tests. Genera with nominal *p* < 0.05 in both CON vs. GLY and ALA vs. GLY comparisons and showing the same direction of change in GLY were defined as candidate genera associated with glycine supplementation. Because this screening was based on nominal *p*-values, these genus-level findings were interpreted as exploratory. The raw 16S rRNA gene sequencing data have been deposited in the NCBI Sequence Read Archive under BioProject PRJNA1520503.

### 2.7. Untargeted Fecal Metabolomics and Data Analysis

Fecal samples collected at D109 were used for untargeted metabolomic analysis (*n* = 10 per treatment), performed by Shanghai Personal Biotechnology Co., Ltd. (Shanghai, China). Each fecal sample (20 mg) was mixed with 300 μL of prechilled methanol containing 5 mg/L L-2-chlorophenylalanine as an internal standard. After vortexing, grinding, sonication, and low-temperature extraction, samples were centrifuged at 12,000 rpm for 10 min at 4 °C, and the supernatants were filtered through 0.22 μm membranes. Equal volumes of filtrate from each sample were pooled to prepare quality-control (QC) samples. QC samples were injected periodically to monitor instrument stability and data quality.

Samples were analyzed using a Vanquish Flex ultra-high-performance liquid chromatography system coupled to an Orbitrap Exploris 120 high-resolution mass spectrometer (Thermo Fisher Scientific, Waltham, MA, USA). Data were acquired separately in positive- and negative-ion modes. Detailed chromatographic and mass spectrometric parameters are provided in the Supplementary Methods. Raw data were processed using MS-DIAL (version 4.9.221218) [31] for peak extraction, alignment, missing-value processing, normalization, and metabolite annotation. Features with >50% missing values in all treatment groups were removed, and remaining missing values were imputed before normalization. Metabolites were putatively annotated using the Personalbio Next-Generation Metabolomics Database. Adenosine, guanosine, and cyclic guanosine monophosphate (cGMP) were putatively annotated by accurate mass and tandem mass spectrometry spectral-library matching, corresponding to Metabolomics Standards Initiative level 2 identification.

Positive- and negative-ion-mode data were preprocessed, annotated, and statistically analyzed separately. For each pairwise comparison, metabolic features were compared using two-tailed independent-samples *t*-tests, and *p*-values were adjusted for multiple testing using the Benjamini–Hochberg false discovery rate (FDR) procedure. Metabolites with nominal *p* < 0.05 in both the CON vs. GLY and ALA vs. GLY comparisons and showing the same direction of change in GLY were retained as glycine-associated candidate metabolites for exploratory analysis.

Candidate metabolites associated with glycine supplementation were matched to compound IDs in the Kyoto Encyclopedia of Genes and Genomes (KEGG) database [32] and subjected to *Sus scrofa* metabolic pathway enrichment analysis. The 297 measured features with *Sus scrofa* KEGG pathway annotations were used as the experiment-specific background. Enrichment was tested using the hypergeometric test, and pathway *p*-values were adjusted using the Benjamini–Hochberg method. The raw and processed untargeted metabolomics data have been deposited in MetaboLights under study accession MTBLS15712.

### 2.8. Correlation Analysis Between Gut Microbiota and Metabolites

Paired gut microbiota and fecal metabolomics data from all 30 sows at D109 were used for Spearman rank correlation analysis. The analysis included the candidate genera *Streptococcus* and CAG-873 and the purine-metabolism-related candidate metabolites adenosine, guanosine, and cyclic guanosine monophosphate (cGMP), yielding six genus–metabolite correlations. Spearman correlation coefficients and nominal *p*-values were reported, and the Benjamini–Hochberg method was applied across the six tests to control the FDR. This analysis was exploratory.

### 2.9. Statistical Analysis

Statistical analyses were performed using SPSS 27.0.1 (IBM Corp., Armonk, NY, USA), and graphs were generated using GraphPad Prism 9.5.1 (GraphPad Software, San Diego, CA, USA). For reproductive performance and early piglet growth outcomes, pregnancy status was recorded as an individual binary outcome for each of the 20 gilts per treatment, with the individual gilt serving as the experimental unit. Overall treatment differences in pregnancy rate were assessed using the Fisher–Freeman–Halton exact test; a significant overall result was followed by pairwise Fisher’s exact tests. For continuous reproductive and litter-level outcomes, including live-born litter weight at birth and litter weight at 28 d, the sow/litter served as the experimental unit. Normality and homogeneity of variance were assessed using the Shapiro–Wilk and Levene tests, respectively. Data meeting these assumptions were compared among the three treatment groups using one-way analysis of variance (ANOVA), followed by Fisher’s least significant difference (LSD) test for pairwise comparisons when the overall treatment effect was significant. The proportions of stillborn and low-birth-weight piglets were analyzed using generalized estimating equations with a binomial distribution and logit link, treatment as the fixed effect, and sow (litter) as the clustering unit to account for within-litter correlation. A significant overall treatment effect was followed by pairwise Wald tests. The one-way ANOVA model was:

$$Y_{ij}=\mu +T_{i}+\varepsilon_{ij}$$

where *Y_ij_* is the observation for the *j*th experimental unit in the *i*th treatment group, *μ* is the overall mean, *T_i_* is the fixed effect of treatment, and *ε_ij_* is the random error.

For placental histological and gene-expression outcomes, the sow was the experimental unit. Data meeting the assumptions of normality and homogeneity of variance were analyzed using one-way ANOVA followed by Fisher’s LSD test when the overall treatment effect was significant. These analyses were considered exploratory. When assumptions were potentially violated, Welch’s ANOVA or the Kruskal–Wallis test was used as a sensitivity analysis, as appropriate.

For gut microbiota analyses, alpha-diversity indices were compared using the Kruskal–Wallis test. LEfSe analyses were performed as described in Section 2.6. At D109, genus-level relative abundances were compared pairwise between groups using two-tailed independent-samples *t*-tests, and the candidate-genus analyses were considered exploratory.

For untargeted metabolomics, positive- and negative-ion-mode data were analyzed separately as described in Section 2.7. Metabolic features were compared using two-tailed independent-samples *t*-tests, with *p*-values adjusted using the Benjamini–Hochberg false discovery rate (FDR) procedure. KEGG pathway enrichment was evaluated using the hypergeometric test, with pathway *p*-values adjusted using the Benjamini–Hochberg method. Spearman rank correlation analysis was used for genus–metabolite correlations as described in Section 2.8, with the Benjamini–Hochberg procedure applied across the six tested correlations.

Pregnancy-rate analysis included 20 gilts per treatment; the remaining reproductive and early piglet growth outcomes included 10 sows per treatment. Placental histology and gene-expression analyses included 5 sows per treatment. D30 gut microbiota analysis included 9 sows per treatment, whereas D109 gut microbiota and untargeted metabolomics analyses included 10 sows per treatment. General continuous reproductive and early piglet growth outcomes are presented as mean ± standard error of the mean (SEM). Relative abundances of candidate genera, normalized metabolite abundances, placental vascular-density data, and gene-expression data are presented as mean ± standard deviation (SD). Unless otherwise specified, nominal statistical significance was set at *p* < 0.05. For analyses using multiple-testing correction, FDR < 0.05 was considered statistically significant.

## 3. Results

### 3.1. Reproductive Performance and Early Piglet Growth

Reproductive performance and early piglet growth outcomes are summarized in Table 2. Pregnancy rate differed among treatments (*p* = 0.004) and was higher in GLY than in CON; ALA did not differ significantly from either CON or GLY.

Total born and born alive showed overall treatment effects (*p* = 0.017 and *p* = 0.009, respectively). Total born was greater in CON and GLY than in ALA, whereas born alive was greater in GLY than in ALA. Neither outcome differed between GLY and CON. Mean individual birth weight did not differ significantly among treatments (*p* = 0.054).

Live-born litter weight at birth and litter weight at 28 d both differed among treatments (*p* < 0.001) and were greater in GLY than in CON and ALA.

The GEE analysis showed a treatment effect on the proportion of low-birth-weight piglets (*p* = 0.010), which was lower in GLY than in both CON and ALA. Stillbirth rate did not differ significantly among treatments (*p* = 0.057).

### 3.2. Placental Vascular Density and mRNA Expression of Related Genes

Representative gross placental appearance and H&E-stained sections are shown in Figure 2A,B. Placental stromal vascular density (PSVD) and placental fold vascular density (PFVD) were greater in GLY than in CON and ALA (*p* < 0.05; Figure 2C,D).

Among angiogenesis- and vascular-function-related genes, placental *VEGFA* mRNA expression was lower in GLY than in CON and ALA, whereas *NOS3* and *KDR* mRNA expression was higher in GLY than in both control groups (*p* < 0.05; Figure 2E–G).

Among nutrient-transport-related genes, *GLUT3* mRNA expression was greater in GLY than in ALA but did not differ from CON, whereas *GLUT4* mRNA expression was greater in GLY than in CON but did not differ from ALA (Figure 2H,I). *SNAT1*, *SNAT2*, *FATP4*, and *CD36* mRNA expression was greater in GLY than in both CON and ALA (*p* < 0.05; Figure 2J–M). Effect estimates with 95% confidence intervals and sensitivity analyses are provided in Table S4.

### 3.3. Maternal Gut Microbiota Composition, Diversity, and Candidate Genera

At D30 and D109, the dominant microbial composition at the phylum and genus levels was generally similar among treatments. Dominant phyla included Firmicutes_D, Firmicutes_A, Bacteroidota, Proteobacteria, and Actinobacteriota, whereas dominant genera included *Streptococcus*, *Lactobacillus*, and *Corynebacterium* (Figure 3A,B). Venn analysis identified 2108 ASVs shared among CON, ALA, and GLY at D30, and 2336 shared ASVs at D109 (Figure 3C,D).

At D30, the Chao1, observed species, Shannon, and Simpson indices did not differ among treatments (*p* > 0.05). At D109, the Simpson index differed among treatments (*p* = 0.034), whereas the other alpha-diversity indices did not (Figure 3E,F). Bray–Curtis PCoA showed extensive overlap in sample distributions among treatments at both sampling times (Figure 3G,H). Genus-level heatmaps showed broadly similar overall abundance patterns among treatments at D30, although some abundance patterns diverged among groups at D109 (Figure 3I,J).

At D30, LEfSe analysis identified several treatment-associated taxa in both the CON vs. GLY and ALA vs. GLY comparisons (Figure S1). These findings indicate that treatment-associated microbial differences were already detectable at this early-gestation post-treatment time point. Because the downstream integrative analyses presented in this study focused on late gestation, candidate-genus screening was subsequently focused on the D109 microbiota data.

At D109, cross-sectional LEfSe comparisons identified treatment-associated taxa in both GLY-related pairwise comparisons. In CON vs. GLY, Streptococcaceae and *Streptococcus* were enriched in GLY, whereas Lactobacillaceae, *Lactobacillus*, and CAG-873 were enriched in CON. In ALA vs. GLY, Streptococcaceae, *Streptococcus*, and Peptostreptococcaceae were enriched in GLY, whereas CAG-873, *Paraprevotella*, and TANB77 were enriched in ALA (LDA score > 2.0, nominal *p* < 0.05; Figure 3K,L).

Genus-level pairwise comparisons further showed that *Streptococcus* relative abundance was greater in GLY than in both CON and ALA, whereas CAG-873 relative abundance was lower in GLY than in both control groups (nominal *p* < 0.05 for each comparison; Figure 3M,N). Based on the screening criterion, *Streptococcus* and CAG-873 were therefore retained as candidate genera associated with glycine supplementation at D109.

### 3.4. Fecal Metabolomic Profiles, Pathway Enrichment, and Genus–Metabolite Correlations

Pairwise comparisons of fecal metabolic features among CON, ALA, and GLY are shown in Figure 4A–C. Using a univariate screening strategy, 29 metabolites showed nominal *p* < 0.05 in both the CON vs. GLY and ALA vs. GLY comparisons and were consistently higher or lower in GLY across both comparisons. Among these, 13 were higher and 16 were lower in GLY (Figure 4D,E; Table S2). None of the 29 candidate metabolites reached FDR < 0.05 in both GLY-related comparisons; therefore, the individual-metabolite findings were considered exploratory. Guanosine remained significant after Benjamini–Hochberg correction only in the CON vs. GLY comparison (FDR = 0.018).

Among the 29 candidate metabolites, 11 were assigned KEGG compound IDs. Putatively annotated adenosine, guanosine, cGMP, and cortisol mapped to *Sus scrofa* metabolic pathways. Adenosine, guanosine, and cGMP mapped to purine metabolism; adenosine also mapped to one-carbon pool by folate, and cortisol mapped to steroid hormone biosynthesis. After Benjamini–Hochberg correction, purine metabolism remained significantly enriched (raw *p* = 0.000726, FDR = 0.002179), whereas one-carbon pool by folate (raw *p* = 0.078779, FDR = 0.118169) and steroid hormone biosynthesis (raw *p* = 0.318376, FDR = 0.318376) were not significant (Figure 4F; Table S3). Because the enrichment analysis was based on nominally selected candidate metabolites, these pathway-level findings were considered hypothesis-generating.

The normalized abundances of the three purine-related candidate metabolites were then compared directly. Putatively annotated guanosine and adenosine were higher in GLY than in both CON and ALA (nominal *p* < 0.01 for each comparison). Putatively annotated cGMP was also higher in GLY than in CON (nominal *p* = 0.040) and ALA (nominal *p* = 0.006; Figure 4G).

Spearman analysis showed positive coefficients between *Streptococcus* and adenosine (ρ = 0.376, nominal *p* = 0.041) and between *Streptococcus* and guanosine (ρ = 0.394, nominal *p* = 0.031). *CAG-873* was negatively correlated with guanosine at the nominal level (ρ = −0.439, nominal *p* = 0.015). Nominal *p*-values for the remaining correlations ranged from 0.051 to 0.433. After Benjamini–Hochberg correction across the six tests, none of the genus–metabolite correlations reached FDR < 0.05 (minimum FDR = 0.077; Figure 4H).

## 4. Discussion

This study evaluated 1.0% glycine supplementation from 30 d before mating to farrowing in local black gilts, using a basal-diet control and an L-alanine isonitrogenous control. The main outcomes included reproductive performance, early piglet growth, and placental vascular and nutrient-transport-related traits, with maternal gut microbiota and fecal metabolomic profiles also characterized in late gestation. The clearest GLY-associated responses were greater live-born litter weight at birth, a lower proportion of low-birth-weight piglets, greater litter weight at 28 d, and altered placental vascular and nutrient-transport-related phenotypes. Late-gestation microbial and metabolic findings provided additional associative evidence related to these responses.

Pregnancy rate was higher in GLY than in CON, whereas ALA was intermediate and did not differ significantly from either group. The 60.0% pregnancy rate in CON was relatively low. All groups were managed under the same housing conditions and underwent the same altrenogest-based estrus-synchronization, estrus-detection, and artificial-insemination procedures, with no treatment-related adverse health events observed. The available data therefore do not identify a specific management or health factor that explains the lower pregnancy rate in CON. However, because only 20 gilts were assigned to each treatment and no a priori power analysis was performed, random between-group variation cannot be excluded, and the relatively low pregnancy rate in CON may have increased the apparent contrast between GLY and CON. Accordingly, this finding should be confirmed in a larger, independently powered study.

Total born and born alive showed overall treatment effects, but neither differed between GLY and CON. In contrast, live-born litter weight at birth was greater and the proportion of low-birth-weight piglets was lower in GLY than in both CON and ALA, whereas mean individual birth weight did not differ significantly among treatments. Thus, the most consistent GLY-associated responses at birth involved litter weight and the distribution of low-birth-weight piglets rather than litter size. ALA showed lower values than CON or GLY for some litter-size outcomes; however, ALA was included as an isonitrogenous control rather than as a physiologically neutral treatment. Accordingly, CON was considered the primary reference for biological efficacy, whereas ALA provided an isonitrogenous comparison to account for additional nitrogen supply. GLY–ALA differences should therefore not be interpreted automatically as glycine-specific effects. Previous studies similarly indicate that gestational amino acid supply can influence reproductive and placental outcomes in sows [24,33]. Litter weight at 28 d was also greater in GLY than in CON and ALA. Because glycine supplementation ended at farrowing, this difference cannot be attributed to direct glycine supplementation during lactation. The greater live-born litter weight at birth and lower proportion of low-birth-weight piglets may have provided a more favorable starting point for preweaning growth, although maternal lactational performance, including mammary development, milk yield, and milk composition, may also have contributed.

The placenta is the key organ linking maternal nutrient supply to fetal growth, and its vascular network provides the structural basis for the exchange of oxygen, nutrients, and metabolic products between mother and fetus [5,6]. Placental stromal and fold vascular densities were greater in GLY than in both CON and ALA, indicating that glycine supplementation was associated with greater placental vascularization. Maternal nutrition can influence placental vascular development. Previous studies in sows and gilts have shown that the dietary methionine-to-lysine ratio can modify placental angiogenesis [24], γ-aminobutyric acid supplementation can improve placental development-related traits [34], sodium sulfate supplementation can increase placental stromal vascular density [35], and ornithine supplementation can increase placental vascular density and piglet birth weight [36]. Placental *VEGFA* mRNA expression was lower in GLY, whereas *KDR* and *NOS3* expression was higher. This pattern indicates that the greater vascular density observed in GLY was not paralleled by *VEGFA* transcription at farrowing. Placental vascular density reflects structural development accumulated throughout gestation, whereas mRNA expression represents the transcriptional state at the time of sampling. In a temporal study of porcine placental development, *VEGFA* expression was higher in mid-gestation and declined on gestation days 85 and 114, while placental capillary area density continued to increase; *KDR* expression also followed a temporal pattern that did not completely parallel *VEGFA* [37]. Thus, lower *VEGFA* expression at farrowing is not inconsistent with greater placental vascular density. *KDR* encodes VEGFR2, a major receptor in VEGF-related vascular signaling, whereas *NOS3* contributes to endothelial NO production and vascular function. Similar responses have been reported during early porcine gestation following L-arginine supplementation, including increased placental VEGFR2 expression and NO/cGMP-related regulation [38]. Together, the higher *KDR* and *NOS3* expression and greater placental vascular density are consistent with differences in VEGF-receptor- and NO-related vascular regulation in GLY.

Placental nutrient-transport-related transcription also showed a relatively consistent response. *SNAT1*, *SNAT2*, *FATP4*, and *CD36* expression was greater in GLY than in both CON and ALA. The SNAT family mediates neutral amino acid transport, whereas *FATP4* and *CD36* are involved in fatty-acid uptake and transport. In contrast, *GLUT3* and *GLUT4* differed only in selected pairwise comparisons, indicating a less consistent glucose-transporter response. Placental vascularization provides the structural basis for nutrient delivery to the maternal–fetal exchange interface, whereas placental transporters mediate substrate transfer toward the fetus [3]. The greater vascular density together with higher expression of several amino acid- and fatty acid-transport-related genes was therefore consistent with the greater live-born litter weight at birth in GLY. Because transporter protein abundance and transplacental nutrient fluxes were not measured, these findings should be interpreted as molecular phenotypes related to nutrient transport rather than direct evidence of increased transplacental nutrient transfer.

Pregnancy-associated changes in the maternal gut microbiota are well recognized [19,20,21], and porcine gut microbial communities are influenced by host genetics, diet, and housing environment [39]. In the present study, the most consistent late-gestation microbial pattern was greater relative abundance of *Streptococcus* and lower relative abundance of CAG-873 in GLY than in both CON and ALA. Dietary interventions during gestation have similarly been associated with changes in maternal gut microbial profiles in sows [22,40,41]. However, the present study does not establish a causal pathway linking glycine-associated microbial differences to reproductive or placental outcomes. *Streptococcus* and CAG-873 should therefore be regarded as candidate microbial features associated with GLY, and their functional relevance requires validation using higher-resolution and intervention-based approaches.

The fecal metabolomic analysis identified purine metabolism as the most prominent pathway associated with GLY. Glycine is a substrate for de novo purine nucleotide synthesis [9], and purine metabolism remained significantly enriched after Benjamini–Hochberg correction. Putatively annotated adenosine, guanosine, and cGMP were also higher in GLY than in both CON and ALA at the nominal level, although evidence for individual metabolites was less robust after multiple-testing correction. The pathway-level finding is therefore consistent with the established biochemical role of glycine in purine synthesis. Maternal interventions involving serine, uridine, or nucleotide supply have also been reported to alter nucleotide- or nutrient-related metabolic profiles and reproductive outcomes in pigs [42,43,44], while purinergic-system components and adenosine receptors are dynamically regulated at the maternal–conceptus interface [45,46].

Adenosine can participate in vascular growth and function [13]. In pigs, gestational supplementation with 5′-adenosine monophosphate or adenosine has been associated with greater placental vascular density and improved neonatal outcomes [14], and adenosine supplementation can increase placental ADORA2A expression and improve placental angiogenesis [47]. Adenosine A2A receptor signaling can activate endothelial nitric oxide synthase and increase NO production [48], while L-arginine supplementation during early porcine gestation has been reported to increase placental VEGFR2 expression, NO synthesis, cGMP concentration, and placental vascular development [38]. Accordingly, the enrichment of fecal purine metabolism and the higher normalized abundances of putatively annotated adenosine and cGMP are biologically compatible with the placental *KDR*/*NOS3* and vascular-density findings observed in GLY. However, these measurements were obtained from different biological compartments, and the fecal metabolomic data do not establish altered purinergic signaling in maternal circulation, uterus, or placenta. Genus–metabolite correlation analysis further identified positive nominal associations of *Streptococcus* with adenosine and guanosine and a negative nominal association of CAG-873 with guanosine, but none remained significant after Benjamini–Hochberg correction. These correlations should therefore be regarded as hypothesis-generating associations rather than evidence that these microbial genera contributed causally to the glycine-associated metabolic changes.

This study has several limitations. Although the number of pregnant gilts retained for follow-up was prespecified and selection within each treatment was random, pregnancy rates differed among treatments, resulting in different proportions of pregnant gilts entering the follow-up cohort. Because pregnancy was a post-treatment outcome, this may have affected comparability among the followed groups, and post-pregnancy findings should therefore be interpreted within the selected pregnant cohort. In addition, basal-diet glycine and alanine concentrations and individual feed intake were not quantitatively determined, preventing precise estimation of total amino acid intake. Placental assessment was based on one randomly selected placenta per sow, and uterine location and the phenotype of the associated piglet were not recorded. These factors may have limited representativeness, and the absence of formal blinding may have introduced observer bias in histological field selection and image quantification.

Several additional limitations relate to mechanistic interpretation and postnatal follow-up. Placental blood flow, nitric oxide concentrations, and purine-related metabolites in maternal circulation or placental tissue were not directly measured; therefore, the proposed relationships between fecal metabolic changes and placental vascular traits should not be interpreted as established mechanisms. At day 28, only litter weight was available, without longitudinal individual body weights or litter size at weaning, so the contributions of individual piglet growth and litter size could not be separated. Mammary traits and milk production were also not assessed and may have contributed to preweaning growth. Finally, metabolite annotations were based on accurate-mass and MS/MS spectral-library matching without authentic-standard confirmation, and the microbiota and metabolomics findings require independent validation.

## 5. Conclusions

Dietary supplementation with 1.0% glycine from 30 d before mating to farrowing was associated with greater live-born litter weight at birth, a lower proportion of low-birth-weight piglets, and greater litter weight at 28 d in local black gilts. Glycine supplementation was also associated with greater placental stromal and fold vascular densities; higher expression of KDR, NOS3, and nutrient-transport-related genes; and late-gestation differences in candidate gut microbial genera together with significant enrichment of fecal purine metabolism. These findings support further evaluation of glycine as a gestational nutritional strategy in local black gilts.

## Figures and Tables

**Figure 1:**
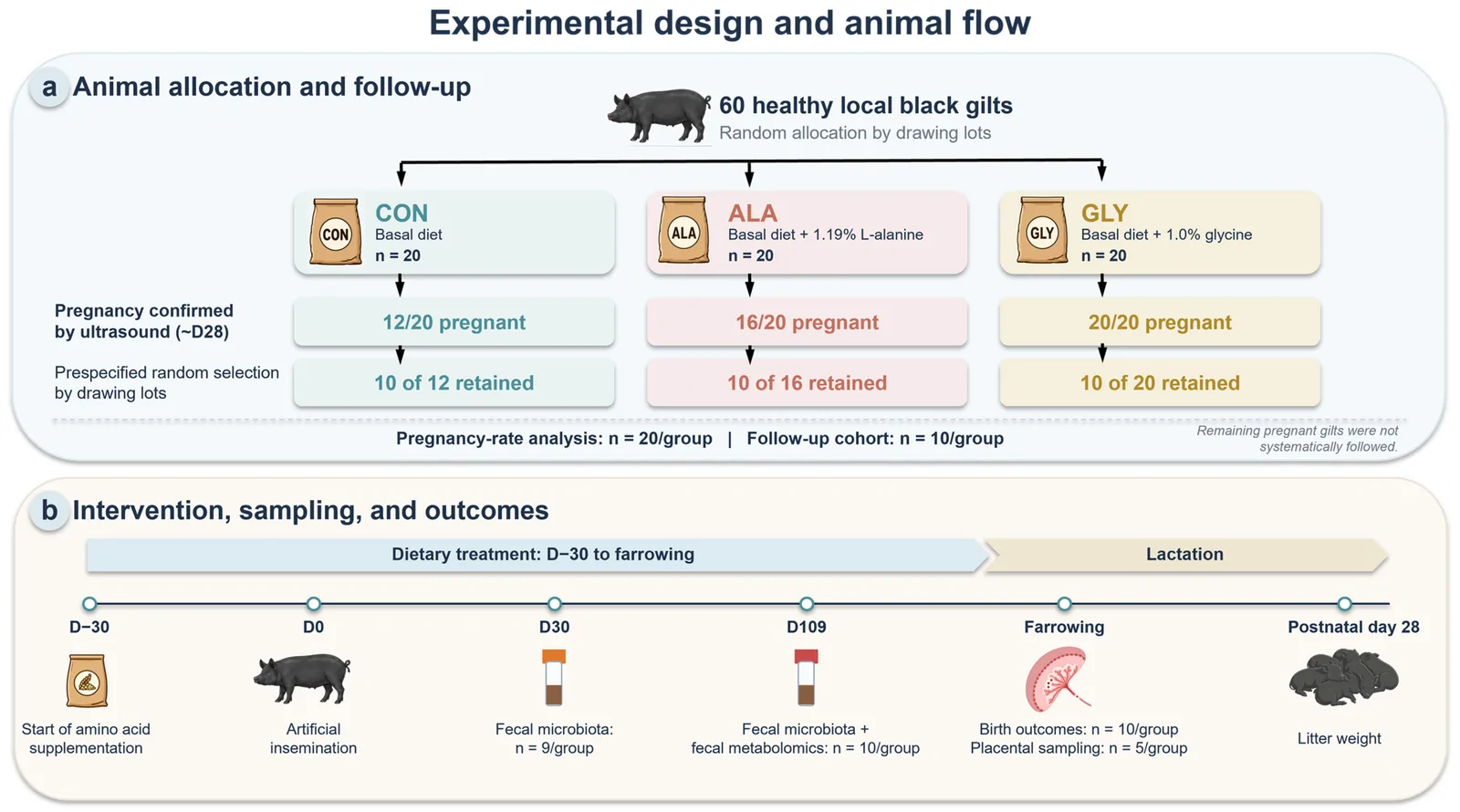
Experimental design, animal flow, and sampling timeline. Pregnancy-rate analysis included all randomized gilts, whereas subsequent follow-up was based on 10 pregnant gilts per treatment. CON, basal diet; ALA, basal diet + 1.19% L-alanine; GLY, basal diet + 1.0% glycine; D-30, 30 d before mating; D0, mating day; D30 and D109, gestation days 30 and 109, respectively.

**Figure 2:**
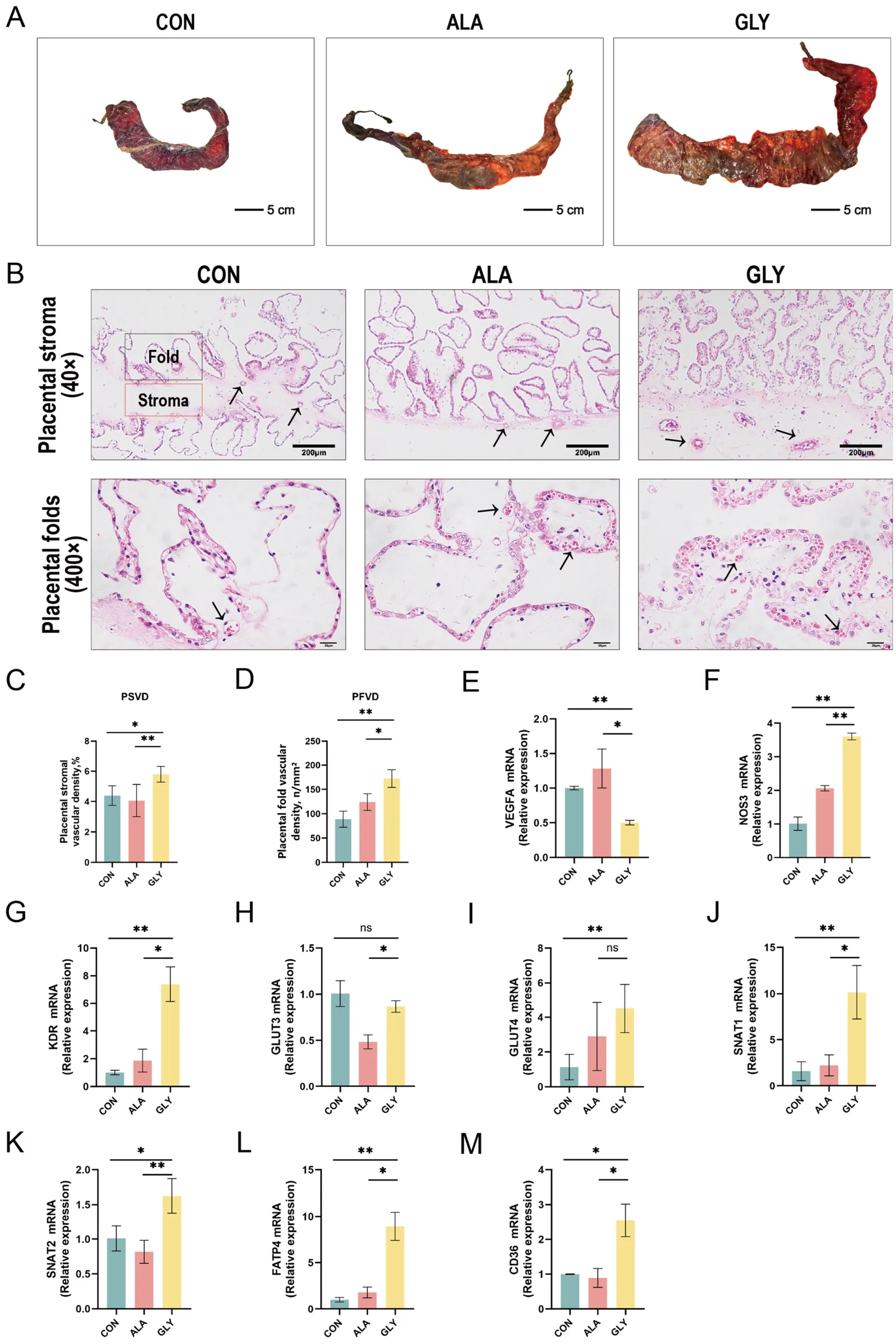
Effects of glycine supplementation from before mating to farrowing on placental vascular density and mRNA expression of genes related to angiogenesis and nutrient transport in local black gilts. (**A**) Representative gross placental appearance. (**B**) Representative H&E-stained placental sections; the upper row shows placental stroma at 40×, and black arrows indicate blood vessels. The lower row shows placental folds at 400×, and black arrows indicate capillaries. (**C**,**D**) Placental stromal vascular density (PSVD) and placental fold vascular density (PFVD). (**E**–**G**) Relative mRNA expression of *VEGFA*, *NOS3*, and *KDR*. (**H**,**I**) Relative mRNA expression of *GLUT3* and *GLUT4*. (**J**–**M**) Relative mRNA expression of *SNAT1*, *SNAT2*, *FATP4*, and *CD36*. Data in (**C**–**M**) are presented as mean ± SD, with *n* = 5 per treatment. Treatment groups were compared using one-way ANOVA followed by Fisher’s least significant difference test for pairwise comparisons when the overall treatment effect was significant. Scale bars: (**A**), 5 cm; (**B**) upper row, 200 μm; (**B**) lower row, 20 μm. * *p* < 0.05; ** *p* < 0.01; ns = not significant. CON = basal diet; ALA = basal diet + 1.19% L-alanine; GLY = basal diet + 1.0% glycine; H&E = hematoxylin and eosin; PSVD = placental stromal vascular density; PFVD = placental fold vascular density.

**Figure 3:**
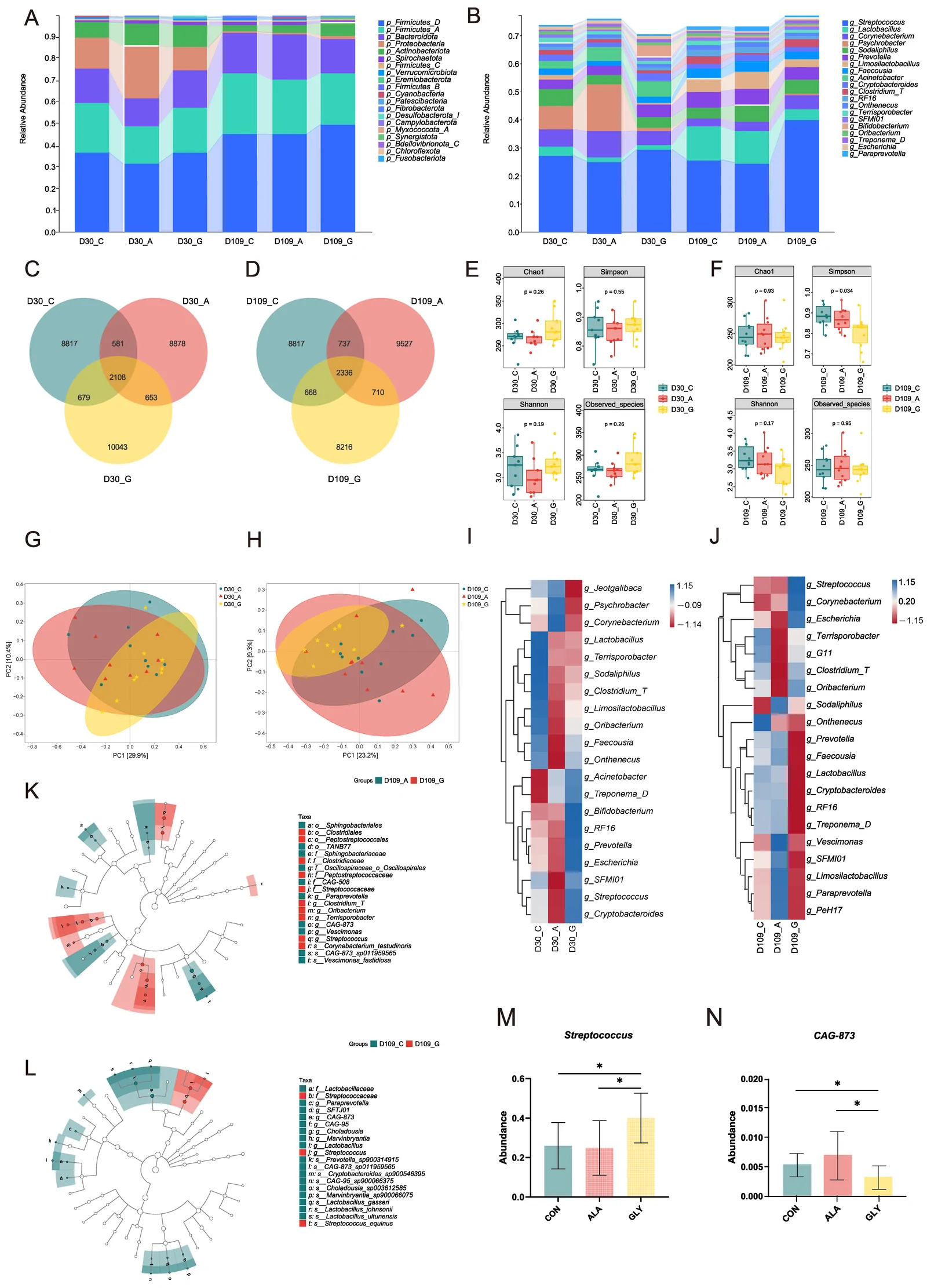
Effects of glycine supplementation from before mating to farrowing on gut microbiota composition, diversity, and candidate genera in local black gilts. (**A**) The 20 most abundant phyla in fecal samples at D30 and D109. (**B**) The 20 most abundant genera at D30 and D109. (**C**,**D**) ASV Venn diagrams at D30 and D109. (**E**,**F**) Alpha-diversity indices at D30 and D109. (**G**,**H**) Principal coordinate analysis (PCoA) at D30 and D109 based on Bray–Curtis distances. (**I**,**J**) Clustering heatmaps of the 20 most abundant genera at D30 and D109. (**K**,**L**) LEfSe analyses of D109 CON vs. GLY and ALA vs. GLY. (**M**,**N**) Relative abundances of *Streptococcus* and CAG-873. Data in (**M**,**N**) are presented as mean ± SD and were compared pairwise using two-tailed independent-samples *t*-tests. The LEfSe screening threshold was LDA score > 2.0 and nominal *p* < 0.05; asterisks in (**M**,**N**) indicate nominal pairwise *p*-values. * *p* < 0.05. CON = basal diet; ALA = basal diet + 1.19% L-alanine; GLY = basal diet + 1.0% glycine; D30 = gestation day 30; D109 = gestation day 109; ASV = amplicon sequence variant; PCoA = principal coordinate analysis; LEfSe = linear discriminant analysis effect size; LDA = linear discriminant analysis. *n* = 9 per treatment at D30 and *n* = 10 per treatment at D109.

**Figure 4:**
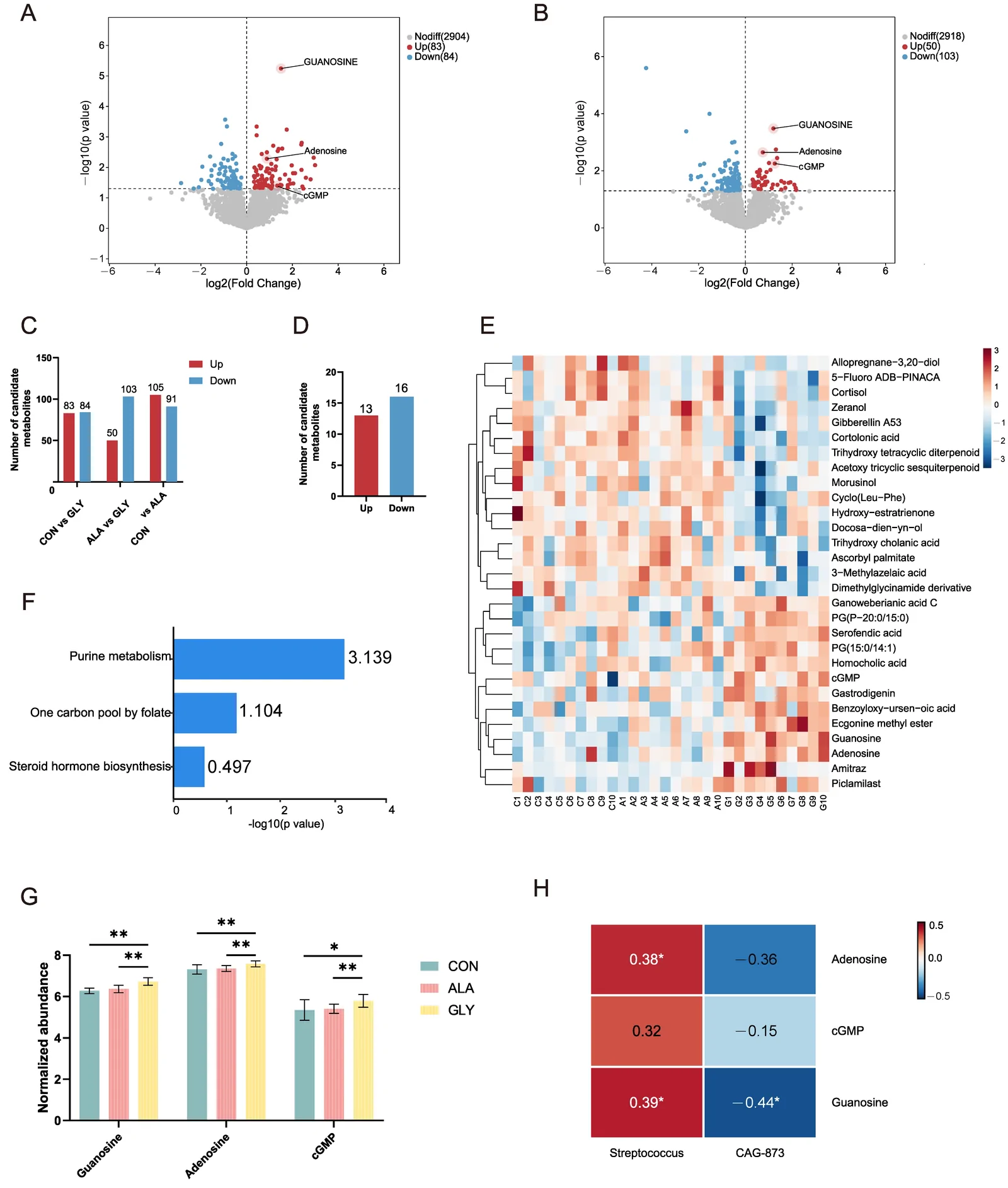
Fecal metabolomic profiles and genus–metabolite correlations on gestation day 109. (**A**,**B**) Volcano plots of metabolic features for CON vs. GLY and ALA vs. GLY, respectively. (**C**) Numbers of differential metabolic features in the three pairwise comparisons. (**D**) Numbers of the 29 glycine-associated candidate metabolites showing higher or lower abundance in GLY. (**E**) Hierarchical clustering heatmap of the 29 candidate metabolites. (**F**) KEGG pathway enrichment analysis. (**G**) Normalized abundances of putatively annotated guanosine, adenosine, and cGMP. (**H**) Spearman correlations of *Streptococcus* and *CAG-873* with the three purine-related metabolites. Data in (**G**) are presented as mean ± SD. Asterisks in (**G**,**H**) indicate nominal *p*-values; FDR-adjusted results are reported in the text. * *p* < 0.05; ** *p* < 0.01. CON, control; ALA, L-alanine; GLY, glycine; KEGG, Kyoto Encyclopedia of Genes and Genomes; cGMP, cyclic guanosine monophosphate. *n* = 10 per treatment.

**Table 1:** Ingredient composition and calculated nutrient composition of the gestation and lactation diets (as-fed basis).

Item	Gestation Diet	Lactation Diet
**Ingredient composition, g/kg**		
Corn	557.35	459.55
Soybean meal (43% CP)	148.05	186.00
Wheat middlings	80.00	80.00
Rice bran meal	60.00	50.00
Rice bran	60.00	–
Wheat bran	45.30	38.80
Wheat flour	–	50.00
Corn DDGS (high-fat)	–	20.00
Soybean hulls	–	20.00
Glucose	–	20.00
Soybean oil	10.00	15.00
Fish meal	–	15.00
Dicalcium phosphate	12.40	10.25
Limestone	11.00	15.40
Sodium chloride	4.50	4.50
0.5% vitamin–mineral premix	5.00 (5H53A) ^1^	5.00 (5H53B) ^2^
L-lysine sulfate (70%)	3.70	–
L-lysine hydrochloride (98%)	–	4.05
Choline chloride (60%)	1.00	1.50
L-threonine	0.85	1.80
L-methionine	0.55	1.25
L-valine	–	1.45
L-tryptophan	0.05	0.10
Mycotoxin adsorbent (brewer’s yeast cell wall)	0.15	0.15
Commercial feed antioxidant (Baoxiankang T60) ^4^	0.10	0.20
Total	1000.00	1000.00
Calculated nutrient composition ^3^, g/kg		
Dry matter	872	875
Crude protein	152	174
Ether extract	47	43
Crude fiber	72	64
Crude ash	78	82
Calcium	8.5	9.2
Total phosphorus	6.2	6.8
Sodium chloride	4.2	5.0
Lysine	10.5	11.4

CP = crude protein; DDGS = distillers dried grains with solubles. ^1^ The 5H53A vitamin–mineral premix was included at 5.0 g/kg of the gestation diet and contributed the following amounts per kilogram of complete diet: Fe, 120 mg; Cu, 15 mg; Zn, 80 mg; Mn, 60 mg; Se, 0.30 mg; vitamin A, 10,000 IU; vitamin D3, 2500 IU; vitamin E, 60 mg; vitamin K3, 3.0 mg; vitamin B1, 2.0 mg; vitamin B2, 6.0 mg; vitamin B6, 2.0 mg; vitamin B12, 0.03 mg; folic acid, 1.50 mg; nicotinamide, 30 mg; and pantothenic acid, 18 mg. ^2^ The 5H53B vitamin–mineral premix was included at 5.0 g/kg of the lactation diet and contributed the following amounts per kilogram of complete diet: Fe, 120 mg; Cu, 15 mg; Zn, 80 mg; Mn, 60 mg; Se, 0.30 mg; vitamin A, 6000 IU; vitamin D3, 2500 IU; vitamin E, 70 mg; vitamin K3, 3.5 mg; vitamin B1, 2.0 mg; vitamin B2, 8.0 mg; vitamin B6, 2.0 mg; vitamin B12, 0.03 mg; folic acid, 1.50 mg; nicotinamide, 35 mg; and pantothenic acid, 22 mg. ^3^ Nutrient values were calculated from the original diet formulation and ingredient-composition data and are reported on an as-fed basis. Premix vitamin–mineral contributions were calculated from the inclusion rates and manufacturer-provided specifications. ^4^ Baoxiankang T60 was a commercial feed antioxidant used in the diet formulation.

**Table 2:** Effects of glycine supplementation from before mating to farrowing on reproductive performance and early piglet growth in local black gilts.

Item	CON	ALA	GLY	*p* Value
Pregnancy rate, %	12/20 (60.0) ^b^	16/20 (80.0) ^ab^	20/20 (100.0) ^a^	0.004
Total born, piglets/litter	11.20 ± 0.81 ^a^	9.00 ± 0.63 ^b^	12.00 ± 0.68 ^a^	0.017
Born alive, piglets/litter	10.20 ± 0.70 ^ab^	8.60 ± 0.65 ^b^	11.70 ± 0.62 ^a^	0.009
Stillbirth rate, %	10/112 (8.9)	4/90 (4.4)	3/120 (2.5)	0.057
Live-born litter weight at birth, kg/litter	11.35 ± 0.82 ^b^	10.36 ± 0.70 ^b^	15.37 ± 0.79 ^a^	<0.001
Mean individual birth weight, kg/piglet	1.15 ± 0.05	1.24 ± 0.06	1.33 ± 0.05	0.054
Low-birth-weight piglets, %	16/102 (15.7) ^a^	12/86 (14.0) ^a^	4/117 (3.4) ^b^	0.010
Litter weight at 28 d, kg/litter	56.05 ± 4.04 ^b^	48.27 ± 2.94 ^b^	72.77 ± 4.17 ^a^	<0.001

CON = basal diet; ALA = basal diet + 1.19% L-alanine; GLY = basal diet + 1.0% glycine. Data are presented as mean ± SEM unless otherwise indicated. Pregnancy rate is presented as pregnant gilts/total gilts (%), and stillbirth and low-birth-weight piglets are presented as event count/total count (%). Different lowercase superscripts within a row indicate *p* < 0.05. Statistical procedures are described in Section 2.9. Pregnancy rate was based on all 20 randomized gilts per treatment; all other outcomes were based on the 10 pregnant gilts per treatment selected for post-pregnancy follow-up.

## Data Availability

The raw 16S rRNA gene sequencing data generated in this study have been deposited in the NCBI Sequence Read Archive (SRA) under BioProject accession number PRJNA1520503. The raw and processed untargeted metabolomics data, including the feature table and metabolite annotation information, have been deposited in MetaboLights under study accession MTBLS15712. Other data supporting the findings of this study are available from the corresponding author upon reasonable request.

## Supplementary Materials

The following supporting information can be downloaded at: https://www.mdpi.com/article/10.3390/ani16192995/s1, Supplementary Methods: detailed chromatographic and mass spectrometric parameters for untargeted metabolomics; Table S1: Primer sequences used for quantitative real-time PCR analysis in porcine placenta; Table S2: Detailed statistics for 29 exploratory candidate metabolites associated with glycine supplementation; Table S3: KEGG metabolic pathway enrichment analysis of glycine-associated candidate metabolites; Table S4: Effect estimates and sensitivity analyses for exploratory placental histological and gene-expression outcomes; Figure S1: LEfSe analysis of fecal microbiota at gestation day 30.
