# Supplementary material for: Effects of Dietary Glycine Supplementation on Reproductive Outcomes and Early Piglet Growth in Local Black Gilts: Associations with Placental Traits and Maternal Gut Microbiota–Metabolome Profiles
Source: Animals (Basel). 2026 Sep 23;16(19):2995. doi: 10.3390/ani16192995 (PMC13663340; doi:10.3390/ani16192995)
Supplement: Supplementary file 1 [file animals-16-02995-s001.zip › animals-4564879-supplementary.pdf]

Effects of Dietary Glycine Supplementation on Reproductive Outcomes and Early Piglet Growth in Local Black Gilts: Associations with Placental Traits and Maternal Gut Microbiota–Metabolome Profiles

Xingfu Chen, Yuening Xing, Dexin Yuan, Mengxian Chen, Ning Xu, Tiansheng Ma, Keming Huang and Suo Li

Supplementary Materials

Supplementary Methods

Detailed LC–MS conditions for untargeted fecal metabolomics.

Fecal extracts were analyzed using a Vanquish Flex ultra-high-performance liquid chromatography system (Thermo Fisher Scientific) coupled to an Orbitrap Exploris 120 high-resolution mass spectrometer (Thermo Fisher Scientific). Chromatographic separation was performed on an ACQUITY UPLC HSS T3 column (100 Å, 1.8 μm, 2.1 × 100 mm) at a flow rate of 0.4 mL/min and a column temperature of 40 °C. The autosampler was maintained at 8 °C and the injection volume was 2 μL. Mobile phase A was water containing 0.1% formic acid and mobile phase B was acetonitrile containing 0.1% formic acid. The gradient was as follows: 0–1.0 min, 5% B; 1.0–4.7 min, 5–95% B; 4.7–6.0 min, 95% B; 6.0–6.1 min, 95–5% B; and 6.1–8.5 min, 5% B.

Mass spectrometric data were acquired separately in positive- and negative-ion modes using data-dependent acquisition under Xcalibur software (version 4.7; Thermo Fisher Scientific) with a heated electrospray ionization (HESI) source. Spray voltages were +3.5 kV and −3.0 kV for positive- and negative-ion modes, respectively. The sheath gas and auxiliary gas were set to 40 and 10 arbitrary units, respectively; the capillary temperature was 320 °C and the auxiliary-gas heater temperature was 300 °C. Full-scan resolution was 60,000 over an m/z range of 70–1000, with AGC Target set to Standard and a maximum injection time of 100 ms. The four most intense precursor ions were selected for MS/MS fragmentation with a dynamic exclusion time of 4 s. MS/MS spectra were acquired at a resolution of 15,000 using higher-energy collisional dissociation at 30% collision energy, with AGC Target set to Standard and maximum injection time set to Auto.

Pooled quality-control (QC) samples were prepared by combining equal aliquots of individual sample filtrates. Two to four QC injections were analyzed before the study samples to equilibrate the analytical system, followed by one QC injection every 6–12 study samples. Raw files were processed using MS-DIAL (version 4.9.221218). The main library-search settings were: MS1 tolerance for identification, 0.01; MS2 tolerance for identification, 0.05; smoothing level, 3; minimum peak height, 10,000; minimum peak width, 5; mass slice width, 0.05; and identification score cut-off, 70.

Table S1. Primer sequences used for quantitative real-time PCR analysis in porcine placenta.

Target gene	**Gene accession**	**Primer**	**Primer sequence (5′–3′)**
VEGFA	NM_214084	Forward	AGGCCAGCACATAGGAGAGA
		Reverse	CGCGAGTCTGTGTTTTTGCA
KDR	AF513909.1	Forward	CAACTTGCAAGACAGTGGCC
		Reverse	TTGAAGTTCGGACATGAGGGC
NOS3	NM_214295.1	Forward	ATCTTCAGCCCCAAACGGAG
		Reverse	AACATTTCCGCCTGTGCAC
GLUT3	XM_021092391.1	Forward	TCTCCATCATGCTCCAGCTCTCC
		Reverse	AATAGTGGCATAGATTGGCTCCTGAAC
GLUT4	NM_001128433	Forward	TATGTTGCGGATGCTATGGG
		Reverse	CTCGGGTTTCAGGCACTTTT
SNAT1	XM_003355629	Forward	AAGAACCTGGGCTATCTCGG
		Reverse	TGTTGCGTTAGGACTCGTTG
SNAT2	NM_001317081.1	Forward	GTTACCTTTGGTGATCCAGGC
		Reverse	ACCAATGACACCAGCAGAACC
FATP4	XM_003353676.1	Forward	AGCCGCATCCTGTCCTTT
		Reverse	GACATCCTTGGCGATCTTTT
CD36	DQ192230.1	Forward	GGACTCATTGCTGGTGCTGT
		Reverse	GTCTGTAAACTTCCGTGCCTGT
ACTB	XM_021086047.1	Forward	GATCTGGCACCACACCTTCTACAAC
		Reverse	TCATCTTCTCACGGTTGGCTTTGG

Table S2. Detailed statistics for 29 exploratory candidate metabolites associated with glycine supplementation.

(A) CON vs GLY

Metabolite	Mode	Direction	KEGG ID	CG FC (95% CI)	CG *p*	CG FDR
Guanosine	NEG	Up	C00387	2.828 (2.004–3.992)	5.699e-06	0.018
PG(15:0/14:1(9Z))	NEG	Up	-	3.365 (1.825–6.203)	5.773e-04	0.355
(-)-Ecgonine methyl ester	POS	Up	C12448	1.353 (1.153–1.588)	8.853e-04	0.453
Adenosine	POS	Up	C00212	1.844 (1.230–2.762)	0.005	0.760
Homocholic acid	POS	Up	-	7.498 (1.867–30.111)	0.007	0.790
3-Benzoyloxy-11-oxo-12-ursen-28-oic acid	POS	Up	-	2.417 (1.278–4.571)	0.009	0.790
serofendic acid	NEG	Up	-	5.262 (1.507–18.373)	0.012	0.790
PG(P-20:0/15:0)	NEG	Up	-	2.113 (1.124–3.972)	0.023	0.884
Gastrodigenin	POS	Up	C17467	3.629 (1.193–11.043)	0.026	0.884
Piclamilast	POS	Up	-	1.584 (1.042–2.409)	0.033	0.884
Amitraz	POS	Up	C10995	2.873 (1.140–7.242)	0.039	0.884
cGMP	NEG	Up	C00942	2.526 (1.049–6.079)	0.040	0.884
Ganoweberianic acid C	NEG	Up	-	1.484 (1.024–2.151)	0.043	0.884
(1S,2R,3R,5S,9S,10S,11S,12R,13R,16R)-3,9,12-trihydroxy-2,6,10,16-tetramethyl-14-oxatetracyclo[11.2.1.02,11.05,10]hexadec-6-ene-8,15-dione	NEG	Down	-	0.729 (0.584–0.909)	0.008	0.790
Cortolonic acid	NEG	Down	-	0.479 (0.281–0.817)	0.010	0.790
5-Fluoro ADB-PINACA	NEG	Down	-	0.442 (0.233–0.841)	0.016	0.832
Zeranol	NEG	Down	C14752	0.850 (0.742–0.974)	0.022	0.884
Cortisol	POS	Down	C00735	0.458 (0.232–0.905)	0.027	0.884
Cyclo(Leu-Phe)	NEG	Down	C20519	0.754 (0.588–0.967)	0.034	0.884
3beta-hydroxy-estra-5,7,9-trien-17-one	NEG	Down	-	0.659 (0.449–0.968)	0.035	0.884
Ascorbyl palmitate	NEG	Down	-	0.586 (0.358–0.962)	0.036	0.884
Morusinol	NEG	Down	C17868	0.616 (0.388–0.978)	0.041	0.884
3alpha,7alpha,11alpha-Trihydroxy-12-oxo-5beta-cholan-24-oic Acid	NEG	Down	-	0.668 (0.454–0.983)	0.041	0.884
(3S,4E,15Z)-Docosa-4,15-Dien-1-Yn-3-Ol	POS	Down	-	0.600 (0.365–0.985)	0.044	0.884
allopregnane-3,20-diol	POS	Down	-	0.563 (0.326–0.973)	0.046	0.884
3-Methylazelaic acid	NEG	Down	-	0.679 (0.470–0.979)	0.046	0.884
Gibberellin A53	NEG	Down	C06094	0.782 (0.613–0.998)	0.048	0.884

Metabolite	Mode	Direction	KEGG ID	CG FC (95% CI)	CG *p*	CG FDR
7,10-bis(acetyloxy)-2-hydroxy-8,11,11-trimethyl-3-methylidenetricyclo[6.3.0.02,6]undecan-4-yl acetate	NEG	Down	-	0.812 (0.661–0.999)	0.049	0.884
N-(1,5-Dimethyl-3-oxo-2-phenyl-2,3-dihydro-1H-pyrazol-4-yl)-N^2,N^2-dimethylglycinamide	NEG	Down	-	0.797 (0.636–0.999)	0.049	0.884

(B) ALA vs GLY

Metabolite	AG FC (95% CI)	AG *p*	AG FDR
Guanosine	2.297 (1.547–3.410)	3.302e-04	0.318
PG(15:0/14:1(9Z))	1.838 (1.085–3.114)	0.026	0.886
(-)-Ecgonine methyl ester	1.244 (1.039–1.489)	0.020	0.886
Adenosine	1.679 (1.236–2.280)	0.002	0.771
Homocholic acid	4.440 (1.083–18.193)	0.039	0.898
3-Benzoyloxy-11-oxo-12-ursen-28-oic acid	2.477 (1.471–4.170)	0.002	0.771
serofendic acid	3.274 (1.064–10.075)	0.040	0.898
PG(P-20:0/15:0)	2.190 (1.038–4.620)	0.041	0.898
Gastrodigenin	2.902 (1.025–8.221)	0.045	0.898
Piclamilast	1.496 (1.058–2.116)	0.025	0.886
Amitraz	3.173 (1.267–7.943)	0.026	0.886
cGMP	2.397 (1.338–4.296)	0.006	0.886
Ganoweberianic acid C	1.465 (1.058–2.030)	0.024	0.886
(1S,2R,3R,5S,9S,10S,11S,12R,13R,16R)-3,9,12-trihydroxy-2,6,10,16-tetramethyl-14-oxatetracyclo[11.2.1.02,11.05,10]hexadec-6-ene-8,15-dione	0.776 (0.646–0.934)	0.010	0.886
Cortolonic acid	0.494 (0.293–0.834)	0.011	0.886
5-Fluoro ADB-PINACA	0.487 (0.267–0.890)	0.022	0.886
Zeranol	0.770 (0.661–0.899)	0.002	0.771
Cortisol	0.499 (0.263–0.948)	0.035	0.898
Cyclo(Leu-Phe)	0.759 (0.580–0.993)	0.045	0.898
3beta-hydroxy-estra-5,7,9-trien-17-one	0.690 (0.507–0.938)	0.021	0.886
Ascorbyl palmitate	0.541 (0.329–0.889)	0.018	0.886
Morusinol	0.601 (0.399–0.905)	0.018	0.886

Metabolite	AG FC (95% CI)	AG *p*	AG FDR
3alpha,7alpha,11alpha-Trihydroxy-12-oxo-5beta-cholan-24-oic Acid	0.623 (0.412–0.942)	0.027	0.886
(3S,4E,15Z)-Docosa-4,15-Dien-1-Yn-3-Ol	0.552 (0.306–0.997)	0.049	0.898
allopregnane-3,20-diol	0.612 (0.375–0.9997)	0.0499	0.898
3-Methylazelaic acid	0.550 (0.380–0.797)	0.005	0.886
Gibberellin A53	0.757 (0.589–0.974)	0.033	0.886
7,10-bis(acetyloxy)-2-hydroxy-8,11,11-trimethyl-3-methylidenetricyclo[6.3.0.02,6]undecan-4-yl acetate	0.797 (0.656–0.969)	0.025	0.886
N-(1,5-Dimethyl-3-oxo-2-phenyl-2,3-dihydro-1H-pyrazol-4-yl)-N^2,N^2-dimethylglycinamide	0.764 (0.633–0.921)	0.007	0.886

CG = CON vs. GLY; AG = ALA vs. GLY; FC = fold change, expressed as the abundance in the latter group relative to that in the former group; FDR = Benjamini–Hochberg false discovery rate; POS = positive-ion mode; NEG = negative-ion mode. Direction indicates the change in GLY relative to the corresponding comparison group. Candidate metabolites were selected using nominal $p < 0.05$ in both GLY-related comparisons, with a concordant direction of change in GLY. The 95% confidence intervals (CIs) for FC were calculated from log10-transformed normalized abundances using a pooled-variance independent-samples *t*-based interval and were subsequently back-transformed to the original fold-change scale. Adenosine, guanosine, and cGMP were putatively annotated with confidence corresponding to MSI level 2; none were confirmed using authentic standards. None of the 29 candidate metabolites reached FDR < 0.05 in both GLY-related comparisons. A dash (–) indicates that no KEGG compound ID was assigned. Mode, direction, and KEGG ID are shown in panel A and also apply to the corresponding metabolites in panel B.

Table S3. KEGG metabolic pathway enrichment analysis of glycine-associated candidate metabolites.

**Pathway**	**Mapped candidate metabolite(s)**	**Candidate hits**	**Background hits**	Raw *p*	**BH-FDR**
Purine metabolism	Guanosine; Adenosine; cGMP	3/4	18/297	0.000726	0.002179
One carbon pool by folate	Adenosine	1/4	6/297	0.078779	0.118169
Steroid hormone biosynthesis	Cortisol	1/4	27/297	0.318376	0.318376

Among the 29 glycine-associated candidate metabolites, 11 were assigned KEGG compound IDs, of which 4 mapped to *Sus scrofa* metabolic pathways and were used as the enrichment input. The experiment-specific background consisted of 297 detected features annotated to *Sus scrofa* KEGG metabolic pathways. Benjamini–Hochberg correction was applied across the three tested pathways. FDR < 0.05 was considered statistically significant.

Table S4. Effect estimates and sensitivity analyses for exploratory placental histological and gene-expression outcomes.

Outcome	CON mean ± SD	ALA mean ± SD	GLY mean ± SD	Overall ANOVA p	GLY–CON mean difference (95% CI)	GLY–ALA mean difference (95% CI)	Sensitivity analysis
PSVD	4.397 ± 0.645	4.400 ± 0.542	5.800 ± 0.516	0.0367	+1.403 (+0.263, +2.544)	+1.400 (+0.260, +2.540)	—
PFVD	88.842 ± 16.381	124.168 ± 16.897	172.462 ± 18.270	0.0030	+83.620 (+49.254, +117.986)	+48.294 (+13.928, +82.661)	Kruskal–Wallis, p = 0.0273
VEGFA	1.000 ± 0.025	1.283 ± 0.281	0.503 ± 0.034	0.0032	-0.497 (-0.825, -0.170)	-0.780 (-1.108, -0.453)	Welch ANOVA, p = 0.0003
NOS3	1.014 ± 0.199	2.064 ± 0.079	3.605 ± 0.099	<0.0001	+2.592 (+2.319, +2.864)	+1.541 (+1.269, +1.814)	—
KDR	1.009 ± 0.162	1.870 ± 0.820	7.390 ± 1.259	0.0002	+6.381 (+4.638, +8.124)	+5.520 (+3.777, +7.263)	—
GLUT3	1.007 ± 0.140	0.485 ± 0.076	0.867 ± 0.063	0.0017	-0.139 (-0.337, +0.059)	+0.383 (+0.185, +0.581)	Kruskal–Wallis, p = 0.0390
GLUT4	0.767 ± 0.064	1.867 ± 0.629	2.672 ± 0.489	0.0067	+1.905 (+0.983, +2.827)	+0.805 (-0.116, +1.727)	Kruskal–Wallis, p = 0.0509
SNAT1	1.606 ± 1.030	2.237 ± 1.131	10.138 ± 2.908	0.0026	+8.533 (+4.742, +12.323)	+7.901 (+4.111, +11.691)	—
SNAT2	1.012 ± 0.181	0.819 ± 0.164	1.625 ± 0.248	0.0064	+0.613 (+0.211, +1.015)	+0.806 (+0.404, +1.208)	—
FATP4	1.022 ± 0.245	1.803 ± 0.577	8.917 ± 1.528	<0.0001	+7.895 (+5.991, +9.800)	+7.114 (+5.209, +9.018)	Welch ANOVA, p = 0.0079; Kruskal–Wallis, p = 0.0509
CD36	1.000 ± 0.007	0.892 ± 0.272	2.549 ± 0.466	0.0010	+1.549 (+0.926, +2.171)	+1.656 (+1.034, +2.279)	—

Values are presented as mean ± SD (n = 5 per group). The primary analysis was one-way ANOVA followed by Fisher's LSD for pairwise comparisons. Mean differences are expressed as GLY minus the corresponding comparison group; 95% confidence intervals were calculated using the pooled residual variance from the primary one-way ANOVA. Shapiro–Wilk and Levene tests were used to assess normality and homogeneity of variance, respectively. Where assumption diagnostics indicated a potential violation, Welch ANOVA and/or the Kruskal–Wallis test was used as a sensitivity analysis, as appropriate. Sensitivity analyses were used to evaluate robustness and did not replace the primary analysis. CON, control; ALA, L-alanine; GLY, glycine; CI, confidence interval; PSVD, placental stromal vascular density; PFVD, placental fold vascular density.

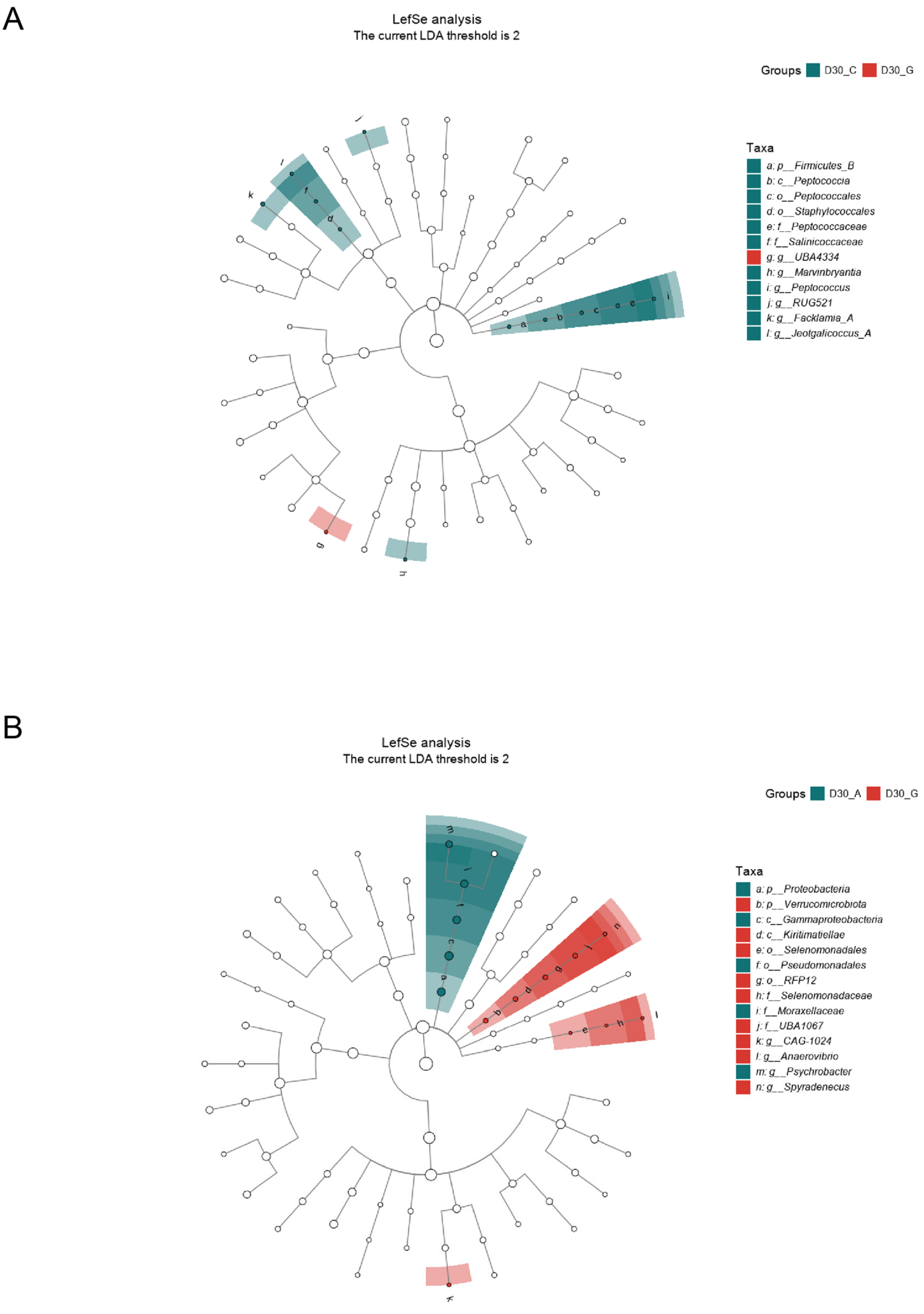

Figure S1. LEfSe analysis of fecal microbiota at gestation day 30. (A) CON vs. GLY and (B) ALA vs. GLY. Differential taxa with LDA scores > 2.0 and nominal p < 0.05 are shown. CON, control; ALA, L-alanine; GLY, glycine.
